# Supplementary material for: Complexome profiling of the Chlamydomonas psb28 mutant reveals TEF5 as an early PSII assembly factor
Source: Plant Cell. 2025 Mar 17;37(6):koaf055. doi: 10.1093/plcell/koaf055 (PMC12164590; doi:10.1093/plcell/koaf055)
Supplement: koaf055_Supplementary_Data [file koaf055_supplementary_data.zip › psb28_interactive.zip!/plots/intensity/qualityPlots/peptides/_Cre04.g217929.t1.1.html]

### Cre04.g217929.t1.1

Trivial name:   
SeqCoverage [median]: 11.20  
Mass: 63.93 kDa  
  
 

| Sequence | MissedCleavage | Mass [Da] | wtIntensity | muIntensity | Score |
| --- | --- | --- | --- | --- | --- |
| AAGLSEDDPAVR | 0 | 1199.58 | 500.76 | 1149.06 | 72.64 |
| KPEVFAILEPYR | 1 | 1460.80 | 513.11 | 0.00 | 43.99 |
| LQVTGEGAR | 0 | 929.49 | 768.76 | 0.00 | 66.69 |
| NHLPVPAVDTEHYR | 0 | 1646.82 | 225.72 | 0.00 | 43.81 |
| SLELSLSELQSR | 0 | 1360.72 | 627.03 | 0.00 | 70.10 |
| VDVSSDGGK | 0 | 862.40 | 0.00 | 488.75 | 66.35 |
|  |  |  | 2635.4 | 1637.8 |  |
